# Recovery of misattributed congenital anosmia after platelet-rich plasma injections: Report of two cases

**DOI:** 10.1016/j.bjorl.2024.101538

**Published:** 2024-11-29

**Authors:** Jérôme R. Lechien, Luigi A. Vaira, Bianca M. Georgescu, Antonino Maniaci, Stéphane Hans, Sven Saussez

**Affiliations:** a University of Mons, EpiCURA Hospital, Department of Otolaryngology-Head and Neck Surgery, Division of Laryngology and Bronchoesophagology, Mons, Belgium; b Université Versailles Saint-Quentin-en-Yvelines (Paris Saclay University), Foch Hospital, School of Medicine, Department of Otorhinolaryngology and Head and Neck Surgery, UFR Simone Veil, Paris, France; c Phoneticsand Phonology Laboratory (UMR 7018 CNRS,Université Sorbonne Nouvelle/Paris 3), Paris, France; d University of Mons (UMONS), Faculty of Medicine, Research Institute for Health Sciences and Technology, Department of Anatomy, Mons, Belgium; e University of Sassari, Department of Medicine, Surgery and Pharmacy, Maxillofacial Surgery Operative Unit, Sassari, Italy; f University of Sassari, PhD School of Biomedical Science, Biomedical Science Department, Sassari, Italy; g Enna Kore University, Medicine and Surgery Department, Enna, Italy

## Introduction

Olfactory Dysfunction (OD) affects 2.7%–24.5% of Western populations.^1^ Most cases of anosmia are attributed to viral infections, trauma, or idiopathic causes^1^ with 1% of anosmia lasting since the birth.^2^ Cases of childbirth anosmia are often considered congenital^2^ including several syndromes (i.e., Kallmann, Laurence-Moon, or Prader-Willi) with an absence of olfactory bulbs. However, in practice, some patients with childbirth anosmia show no anatomical abnormalities and genetic testing is unremarkable, leading to an unclear origin of anosmia. Until recently, there was no effective treatment for long-lasting anosmia. Recent studies have supported the usefulness of the injection of Platelet-Rich Plasma (PRP) in the olfactory clefts of patients with OD. The rationale was based on the regenerative mechanisms of PRP, which includes growth factors, endothelial factors, and anti-inflammatory molecules acting locally in regenerating injured tissues.^1^

In this paper, we present two patients with childbirth anosmia, initially misattributed as congenital, who progressively recovered their sense of smell after (PRP) injections into the olfactory clefts.

## Case 1

**Fig. 1:**
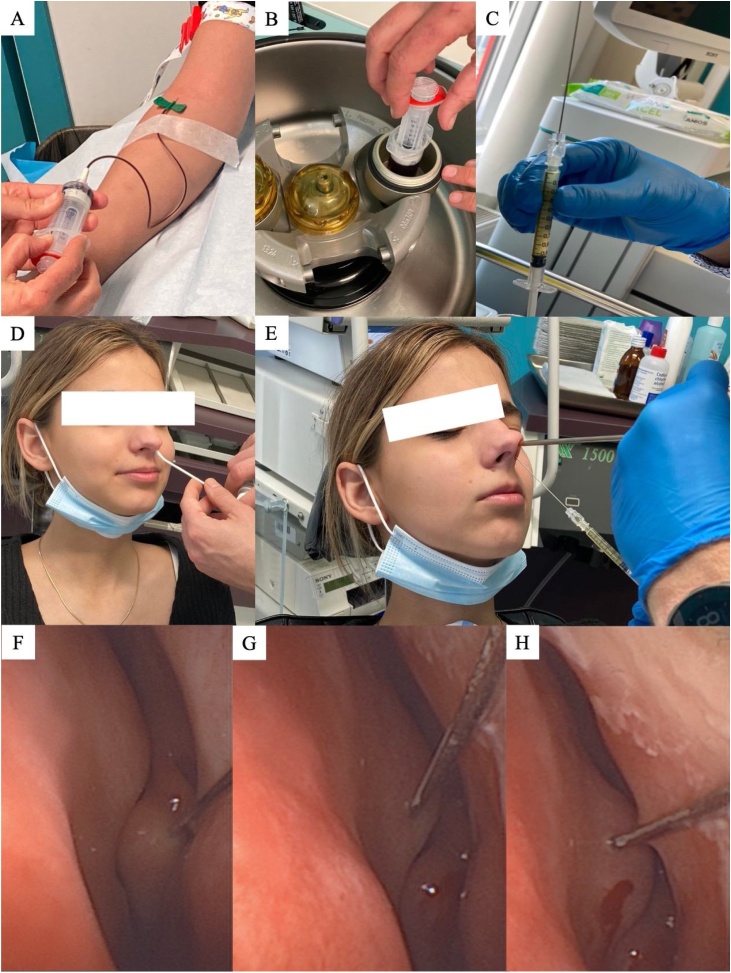
Steps of injection of platelet-rich plasma into the olfactory clefts. The following steps of the procedure are described: (A) Blood extraction into a tube with sodium citrate anticoagulant; (B) Centrifugation at 4200 rpm; (C) Transfer of the PRP into a 1 mL syringe and preparation of the 27-G needle for the injection; (D) Local anesthesia with Xylocain 10% spray 2-minutes after the injection of xylometazoline chlorhydrate drops into the nasal fossae; (E) The injection of the PRP through a bended needle with a 0° rigid optic; (F) Injection of PRP into several points of 0.2–0.5 mL in the nasal septum in regard of the head of the middle turbine, and in the anterior part of the region (G-H).

A 13-year-old male with a childbirth anosmia presented to the otolaryngological consultation of the Dour Medical Center (Belgium). The child reported that he never smelt. The medical and surgical histories reported several rhinitis and otitis media episodes during the first year of life. The nasofibroscopic examination, including an examination of olfactory clefts, was normal. The sinus tomodensitometry was unremarkable. The olfactory bulbs and sulci were reported at the magnetic resonance imaging. The olfactory bulbs reported a reduced volume but were present. Before the consultation, the patient underwent two psychophysical olfactory evaluations at 6 and 12 years old with the Threshold, Discrimination, and Identification (TDI) tests, both reporting complete anosmia (1/48–1/16 at the threshold test, 0 at the discrimination and identification). In August 2023, the patient underwent bilateral PRP injection into the olfactory clefts according to a standardized approach that is described in Fig. 1.^4^ In summary, the procedure includes i) The blood extraction into a tube with sodium citrate anticoagulant; ii) A centrifugation at 4200 rpm; iii) The transfer of the PRP into a 1 mL syringe and preparation of the 27-G needle for the injection; iv) A local anesthesia with Xylocain 10% spray 2 min after the injection of xylometazoline chlorhydrate drops into the nasal fossae; v) The injection of the PRP through a bent needle with a 0° rigid optic; and vi) The injection of PRP into several points of 0.2–0.5 mL in the nasal septum in regard of the head of the middle turbine, and the anterior part of the region (Fig. 1). In the first case, the otolaryngologist injected 2 mL in the right side and 1.0 mL in the left side. The lower volume of the left side was related to partial septum deviation on this side. There was no adverse event throughout the post-injection follow-up. Three weeks after the injection, the child reported being able to detect some odors (lemon, orange, fish, cheese) without being able to recognize them according to the childbirth’s inability to identify odors. The 6-month post-injection TDI score was 15/48 (T:11, D:2, I:2), with a significant improvement of the detection ability of odor (T). An intensive smell rehabilitation was started with a speech therapist to learn to recognize odor stimuli. The child completed the Olfactory Disorder Questionnaire (ODQ), which improved from baseline to 6-month post-injection (54–28). The history of the patient suggested a post-viral long-lasting OD that started in early childhood.

## Case 2

The practitioner performed bilateral PRP injections in a 55-year-old man with a history of complete childbirth anosmia at the TDI (1/48). The patient has never smelt as well. The MRI was normal. The preparation of PRP is standardized. Thus, the preparation of PRP in this case is similar to the first reported case. The medical and surgical histories, nasofibroscopic examination, and the sinus CT-scan were unremarkable. The brain MRI reported an atrophy of both olfactory bulbs. The patient underwent a pre-injection TDI confirming complete anosmia (T:1, D:0, I:0), and the pre-injection ODQ was 65. Several points of 0.3–0.5 mL were performed in the nasal septum in regard of the head of the left middle turbinate (1.3 cc). The procedure was similarly carried out in the right nasal fossae (1.8 cc). The difference between both nasal injections was related to partial septum deviation, limiting the injection of additional PRP on the left side. Similar common precautions were taken to ensure no injection intravascularly, whereas patients were awake during the procedure about any visual changes occurring during the procedure. There was no adverse event. The patient was instructed to follow olfactory training despite several histories of unsuccessful olfactory training sessions. The pre- to 6-month post-injection TDI increased from 1 to 5, while the patient reported progressively smelling some daily life odors 4 weeks after the injection, including smoke, cheese, and citrus. At 10 months, the TDI was 3, 4, 0, highlighting a progressive but slow recovery of the ability to detect and discriminate odors. The post-injection ODQ was 50, reporting a 23% improvement. Importantly, the patient recognized smelling daily life odors, which are not tested within the psychophysical olfactory tests (e.g., fragrance, spicy, toothpaste, cheese or shower gel).

## Discussion

To date, patients with presumed congenital anosmia rarely benefit from a genetic work-up, while the therapeutic arsenal is still inexistent.^1^, ^2^ Recent controlled and randomized studies have shown that injections of Platelet-Rich Plasma (PRP) into the olfactory clefts may be an effective approach for treating longlasting post-viral OD.^3^, ^4^, ^5^ Thus, a randomized controlled trial comparing olfactory cleft PRP injection versus placebo reported an odd ratio of 12.5 in favor of PRP 3-months after the injection in COVID-19 patients.^3^ In a multicenter controlled study, the mean scores of TDI and ODQ significantly improved from pre- to 4-month post-PRP injection in a group of patients with 6-month to 2.5-year history of OD, while the changes of TDI and ODQ were lower in the control group.^4^ Interestingly, the usefulness of PRP was supported in patients with a 4-to-20-year long-lasting post-viral OD, which supported the hypotheses proposing that the long-lasting OD is related to the persistence of virus antigen into the neuroepithelium cells, leading to a long-lasting chronic inflammation in the olfactory cleft, and a related immune reaction.^5^ This hypothesis was additionally supported by authors who identified in experimental animal studies that some viruses, such as Sendai virus or para-influenza viruses, may persist into the olfactory neuroepithelium and/or bulb cells over time.^6^ The Sendai virus persistence is associated with impairment of the ability of olfactory sensory neurons to take up calcium ions after stimulation by suppressing apoptosis of olfactory sensory neurons, which alters the normal regenerative ability of the olfactory epithelium over the long term.^6^ This study corroborated the findings of Mori et al. who detected virus nucleoprotein gene in olfactory bulb cells of infected mice at least 168 days post-infection through polymerase chain reaction.^7^ Regarding the effectiveness of PRP in long-lasting post-viral OD, the findings of the present study may suggest that both patients had a long-lasting (childbirth) post-viral anosmia rather than a true congenital anosmia associated with the inability to develop an effective neuroepithelium. Indeed, in congenital anosmia, the neural tissue associated with the recognition of odor is not developed, while in childbirth anosmia, it is conceivable that the child has developed the brain area when exposed to stimuli. The anti-inflammatory and regenerative properties of PRP may have decreased the inflammatory reaction and induced the regenerative processes, which were impaired for a long time.^3^, ^8^

The primary strength of this paper is its originality in providing olfactory improvements in patients with childbirth anosmia without major anatomical abnormalities on imaging. The recent development of PRP procedures in patients with OD may potentially lead to investigations of new indications of PRP in the olfactology field. The lack of use of objective testing, such as olfactory event-related brain potentials, for documenting the anosmia pattern is the primary limitation. Future studies could evaluate olfactory event-related brain potentials before and after injection of PRP, while biopsies of olfactory clefts could make sense in the analysis of the inflammatory biomarkers and the cell populations. We used both psychophysical olfactory tests and patient-reported outcome questionnaires, but they are both subjective and ODQ could be inadequate for childbirth anosmia. Indeed, this questionnaire mainly assesses the impact of smell loss or distortion on the quality of life of patients. Patients with childbirth anosmia cannot evaluate the negative consequences of smell changes in their daily life because they never smelt.

## Conclusion

To the best of our knowledge, this paper is the first one dedicated to exploring the usefulness of PRP for treating childbirth anosmia. The rationale for publishing these cases was to draw the attention of otolaryngologists to the possibility of attempting PRP injections in patients with childbirth anosmia without the identification of genetic pattern or absence of an olfactory bulb. Future studies are needed to confirm the interest of PRP for childbirth OD without genetic etiology or anatomical abnormalities. Studies could evaluate additional regenerative molecules, such as insulin,^9^ in investigating the local regenerative processes.

## Conflicts of interest

The authors declare no conflicts of interest.
